# Refining the risk for fragile X–associated primary ovarian insufficiency (FXPOI) by *FMR1* CGG repeat size

**DOI:** 10.1038/s41436-021-01177-y

**Published:** 2021-04-29

**Authors:** Emily Graves Allen, Krista Charen, Heather S. Hipp, Lisa Shubeck, Ashima Amin, Weiya He, Sarah L. Nolin, Anne Glicksman, Nicole Tortora, Bonnie McKinnon, Katharine E. Shelly, Stephanie L. Sherman

**Affiliations:** 1 Department of Human Genetics, Emory University School of Medicine, Atlanta, GA USA; 2 Department of Gynecology and Obstetrics, Emory University School of Medicine, Atlanta, GA USA; 3 Department of Human Genetics, New York State Institute for Basic Research in Developmental Disabilities, Staten Island, NY USA

## Abstract

**Purpose:**

Approximately 20–30% of women with an *FMR1* premutation experience fragile X–associated primary ovarian insufficiency (FXPOI); however, current risk estimates based on repeat size only identify women with the midrange of repeats to be at the highest risk.

**Methods:**

To better understand the risk by repeat size, we collected self-reported reproductive histories on 1,668 women and divided them into high-resolution repeat size bins of ~5 CGG repeats to determine a more accurate risk for FXPOI in relation to CGG repeat length.

**Results:**

As previously reported, women with 70–100 CGG repeats were at the highest risk for FXPOI using various statistical models to compare average age at menopause and risk of FXPOI, with women with 85–89 repeats being at the highest risk. Importantly, women with <65 repeats or >120 repeats did not have a significantly increased risk for FXPOI compared to women with <45 repeats.

**Conclusion:**

Using a large cross-section study on 1,668 women, we have provided more personalized risk assessment for FXPOI using high-resolution repeat size bins. Understanding the variability in risk has important implications for family planning and overall health among women with a premutation.

## INTRODUCTION

The 5’ untranslated region of the *FMR1* gene contains a CGG repeat that is responsible for at least three distinct clinical phenotypes: fragile X syndrome (FXS), fragile X–associated tremor/ataxia syndrome (FXTAS), and fragile X–associated primary ovarian insufficiency (FXPOI). The full mutation form of the *FMR1* gene contains more than 200 CGG repeats that are hypermethylated, resulting in transcriptional silencing of the gene.^1^ The lack of *FMR1* protein product, FMRP, which is an RNA-binding protein involved in control of translation,^2^ leads to FXS.^3^ FXTAS and FXPOI are seen in individuals with the premutation form of the *FMR1* gene that contain 55–199 CGG repeats.^4,5^ FXTAS is characterized by intention tremor and gait ataxia, with associated features of parkinsonism, neuropsychological dysfunction, autonomic dysfunction, and peripheral neuropathy.^6^ The prevalence is approximately 40% and 6–18% among men and women, respectively.^7,8^ Women with FXPOI develop hypergonadotropic hypogonadism and have absent or irregular periods prior to age 40.^9^ FXPOI occurs in approximately 20% of women with a premutation, and women with FXPOI have an increased risk of infertility and hypoestrogenism, resulting in hot flashes, night sweats, and increased risk for osteoporosis.^10^ More recently, an additional classification of FXAND (fragile X–associated neuropsychiatric disorders) was proposed to describe an increased frequency of mental health problems, immune disorders, and various other medical conditions among women with a premutation.^11^

While FXPOI is the most frequent of the fragile X–associated disorders, it is underdiagnosed and biologically the least well understood. Because premutation alleles are seen in about 1 in 151 women,^12^ more than 1 million women in the United States carry a premutation allele. In qualitative interviews with women with a premutation, the diagnosis of FXPOI was delayed in women with known premutation status when presenting with symptoms of POI; this delay in diagnosis was even greater for women who did not know their premutation status.^13^ Individuals with premutation alleles and stakeholder groups alike (e.g., health-care providers) have emphasized the importance of understanding risk for FXPOI and identifying predictive markers.^14–17^ The need for this effort becomes magnified as fragile X screening studies become more prevalent and identify women with premutation alleles who are at risk for FXPOI.^14,18–21^

Previous studies have consistently identified that the risk for FXPOI is associated with CGG repeat size in a nonlinear fashion: those with ~70 to 100 CGG repeats are at the highest risk.^10,22–26^ Because the risk is nonlinear, the molecular mechanism causing FXPOI may be distinct from the mechanism for FXTAS, which shows a linear risk with repeat size. However, because the underlying mechanism of this shortened reproductive lifespan is unknown and there are no biomarkers for prediction, all individuals with premutation alleles are “affected” in the sense that they are at risk and no fertility preserving interventions can be prescribed.

In the current work, we examined fragile X repeat sizes among 1,668 women, using high-resolution bins, to determine a more accurate risk for FXPOI in relation to CGG repeat length. We divided repeat sizes into ranges of ~5 repeats where the sample size was adequate, and statistically modeled the risk for an earlier onset of menopause or increased risk for FXPOI for each repeat size group. In addition, we investigated self-reported cycling and fertility characteristics for women in each of these repeat size groups. Our goal is to provide women more personalized risk information based on their repeat size.

## MATERIALS AND METHODS

### Study population

Emory University Institutional Review Board approved protocols and consent forms, and informed consent was obtained from all participants. Participants were identified through two different sampling schemes: (1) a general population survey in metropolitan Atlanta to identify individuals with smaller allele sizes^27^ and (2) from fragile X family recruitment efforts to identify individuals with larger alleles (e.g., previous fragile X research projects at Emory, recruitment at scientific conferences, and through collaborations with other research groups). Once a family contact was identified, additional family members were screened for eligibility without respect to phenotype. Eligibility was based on premutation allele status, age, and sex. A blood or saliva sample was collected, and each participant completed a reproductive history questionnaire. Data included general demographics (e.g., age at interview, date of birth, race/ethnicity), lifestyle factors that might affect overall health (e.g., smoking, body mass index [BMI]), and reproductive history (e.g., menstrual history, reason for cessation of menses, pregnancy history). A subset of women were also interviewed by a reproductive endocrinologist (*n* = 83).^13^ For many women, the questionnaires and interviews were administered at multiple time points. Research personnel curated all data points to determine correct assignments for study variables.

### Variable definitions

#### Age at menopause (AAM)

The reproductive history was used to determine whether a woman was still cycling or why her menses had ceased. If the date of last menstrual period was more than two months prior to the interview, we identified the cause of menses cessation. Diagnosis was based on self-reporting. AAM was assigned if menses had been absent for at least 12 months or 4–6 months if a menopausal-level follicle-stimulating hormone level was available.^28^ If AAM from the in-depth interview differed from information provided on the reproductive questionnaire, the data from the in-depth interview were used, as the interviewer (H.S.H.) had information from all previous reproductive questionnaires during the interview. Thus, she was able to obtain the necessary information to determine the most accurate AAM. Women whose menses ceased because of surgery, pregnancy, chemotherapy or radiation, eating disorders, or hormone use were not assigned an AAM.

#### FXPOI

Women were defined as having FXPOI if their age at natural menopause (AAM) as defined above was < age 40. Although we recognize that experiencing POI and menopause is not the same, we will use the notation of AAM to reference the onset of these conditions for all women. Women who had menopause or were still having menstrual cycles at age 40 or later were classified as not having FXPOI. For some women, a FXPOI assignment could not be made (e.g., women who were still cycling, but younger than age 40; women who had surgery, such as a hysterectomy, before age 40, etc.).

#### Cycle characteristics

For women with more than one questionnaire completed, the answer from their earliest questionnaire was used for age at menarche. For all other cycling traits, we used the questionnaire done in closest time proximity to when the cycles ended. Variables (e.g., short cycles and bleed length) were categorized as described in our previous study:^10^ short cycles were defined as <27 days from the first day of one menstrual period to the first day of the next menstrual period, and short bleed length was defined as less than five days from the time that bleeding began until it completely stopped within an average cycle during the last year they were naturally cycling.

#### Fertility characteristics

Two questions were analyzed to assess fertility problems. First, each woman reported whether she ever had unprotected intercourse for a year or more without getting pregnant. Second, each woman was asked if she had ever visited a doctor, clinic, or hospital because she was unable to become pregnant.

#### Environmental factors

Smoking characteristics were curated across questionnaires when necessary. Variables for having ever smoked regularly (at least one cigarette per day), current smoker, and pack years were determined for each woman. Pack years was used in the final models because it showed the strongest association with AAM. BMI was also calculated based on the earliest questionnaire completed. A binary variable for self-reported race/ethnicity was used due to the vast majority self-reporting as Caucasian (1 = Caucasian; 0 = other or not reported).

### Laboratory methods

DNA was extracted from biological samples using Qiagen Qiamp DNA Blood Mini Kit, Gentra Puregene extraction kit, or prepIT-L2P protocol from Oragene.

FRAXA CGG repeat numbers were determined by a fluorescent sequencer method.^29^ For females with only one allele, a second polymerase chain reaction (PCR) protocol was used to look for larger allele.^30^ If only one allele was seen using this method, the woman was classified as homozygous for that repeat size. The PCRs for FRAXA consisted of 1X PCR Buffer (Gibco/BRL), 10% dimethyl sulfoxide (DMSO), 370 µM deazaG, 500 µM d(ACT), 0.3 µM each primer, 15 ng T4 gene 32, and 1.05 U Roche Expand Long *Taq*. Primers for the FMR1 gene were C: 5’GCTCAGCTCCGTTTCGGTTTCACTTCCGGT3’, and F: 5’AGCCCCGCACTTCCACCAGCTCCTCCA3’.^31^

For a subset of samples (see Supplementary Figure 1), *FMR1* repeat sizing was performed by PCR analysis using AmplideX PCR CE/*FMR1* (Asuragen, Inc.) assay. Repeat size and the presence of repeat size mosaicism (PM/PM: more than one premutation size allele detected; PM/FM: both PM and FM alleles detected) were determined using AmplideX PCR CE/*FMR1* at the New York State Institute for Basic Research in Developmental Disabilities or Xpansion Interpreter (Asuragen, Inc.) at Asuragen.

### Statistical analysis

Standard statistical methods were used. Age, BMI, and smoking pack years are reported as the mean ± standard error. Differences in demographics among repeat size groups were examined using an analysis of variance (ANOVA) model. Differences for binary variables (e.g., race/ethnicity and smoking) among groups were tested using a χ^2^ analysis. ANOVA models to compare means of continuous measures (e.g., AAM) between repeat groups and significant differences between groups were determined using Tukey’s post hoc analysis to control for multiple testing. Logistic regression models were used to test for associations between repeat groups and risk of FXPOI, cycling, and fertility traits. Models included all repeat classes as predictor variables, along with covariates. Women with <45 repeats were the referent group. To compare the AAM across repeat size groups, survival analyses were used. For this analysis, women who had experienced AAM were included as those with the “event,” and the AAM was used as the “event” age. Women who did not have an assigned AAM were censored at the following time points: women who were still cycling and not currently on hormone medication were censored at their age of interview, women who had a surgery that led to cessation of menses (e.g., hysterectomy) were censored at the age of surgery, women who were currently on hormone medication were censored at the last age they were not on hormone medications, and women who reported their periods had stopped for other reasons (e.g., chemotherapy) were censored at the age of their last period. All models were adjusted for age at interview, race/ethnicity, smoking pack years, and BMI.

Linear regression models were used to test for the association of repeat size mosaicism with AAM. In this model the outcome measure was AAM, and the model included repeat size, the quadratic of repeat size, binary variables for the presence or absence of each type of mosaicism (i.e., PM/PM or PM/FM (1 = mosaicism detected; 0 = no mosaicism detected]), age at interview, race/ethnicity, smoking pack years, and BMI.

Because some of the women were related, we confirmed our findings using generalized estimating equations (GEE) and frailty analysis to adjust for the dependency of related individuals.

All analyses were done using SAS 9.4 and R.

## RESULTS

Genotyping results for the *FMR1* CGG repeat were used to group the 1,668 women into repeat size groups. Most repeat size groups included a range of five repeats, with the exception of the <45 repeats group, the 45–54 repeats group, and the higher end of the premutation size range. We collapsed these groups due to decreased sample sizes of either the number of women with known AAM (45–54 repeats group) or overall sample size (higher premutation groups). Table 1 shows the sample size for each of the groups as well as demographic, environmental, and descriptive variables. There were significant differences in the age at interview and race/ethnicity across groups; therefore, all statistical models were adjusted for these variables. Although we did not identify a significant difference between groups for smoking or BMI, these variables were also included in all statistical models based on their known association with AAM.^32,33^

**Table 1:** Demographic, environmental, and descriptive characteristics for repeat size groups.

Repeat group	*N*	Age at interview mean ± S.E.	Race			BMI^a^ mean ± S.E.	% Ever smoke	Pack years mean ± S.E.
			% Caucasian	% African American	% Other/not stated			
<45 repeats	634	43.9 ± 0.6	77.8	13.9	8.3	27.0 ± 0.3	34.0	4.9 ± 0.5
45–54 repeats	66	40.9 ± 1.8	75.8	19.7	4.5	26.8 ± 0.8	37.9	4.6 ± 1.4
55–59 repeats	50	42.8 ± 2.1	88.0	8.0	4.0	26.1 ± 0.9	32.0	2.4 ± 0.7
60–64 repeats	38	47.6 ± 2.5	89.5	0	10.5	26.9 ± 0.9	42.1	6.5 ± 2.1
65–69 repeats	53	51.1 ± 1.8	92.4	3.8	3.8	27.5 ± 0.8	46.1	7.5 ± 1.6
70–74 repeats	73	49.5 ± 1.4	90.4	1.4	8.2	28.8 ± 0.8	31.5	7.3 ± 2.0
75–79 repeats	112	47.4 ± 1.3	88.4	4.5	7.1	26.3 ± 0.5	30.6	3.9 ± 0.9
80–84 repeats	110	48.4 ± 1.3	88.2	3.6	8.2	25.7 ± 0.5	37.4	6.6 ± 1.5
85–89 repeats	117	44.1 ± 1.1	85.6	5.1	9.3	27.8 ± 0.7	34.2	5.3 ± 1.1
90–94 repeats	119	46.3 ± 1.1	89.1	3.4	7.5	26.9 ± 0.6	30.8	4.9 ± 1.1
95–99 repeats	89	44.1 ± 1.3	92.1	2.2	5.7	28.2 ± 0.9	37.9	4.4 ± 1.1
100–104 repeats	62	44.1 ± 1.5	93.5	3.2	3.3	26.6 ± 0.8	29.0	2.4 ± 0.7
105–109 repeats	28	45.5 ± 2.5	92.9	3.6	3.5	27.4 ± 1.3	25.9	1.3 ± 0.6
110–119 repeats	45	43.4 ± 1.9	88.9	4.4	6.7	27.2 ± 1.0	50.0	5.6 ± 1.4
120–129 repeats	35	44.0 ± 2.2	91.4	2.9	5.7	26.5 ± 1.2	31.4	2.5 ± 1.0
130–199 repeats	37	39.0 ± 2.1	94.6	0	5.4	26.9 ± 0.9	29.7	2.4 ± 0.9
Total	1668	45.0 ± 0.3	84.6	8.1	7.3	27.0 ± 0.2	34.5	4.9 ± 0.3

*BMI* body mass index.

^a^For women with multiple questionnaires, answer from first questionnaire was used.

### Risk for AAM/FXPOI based on repeat size group

#### Adjusted mean AAM per repeat size group (ANOVA)

**Fig. 1:**
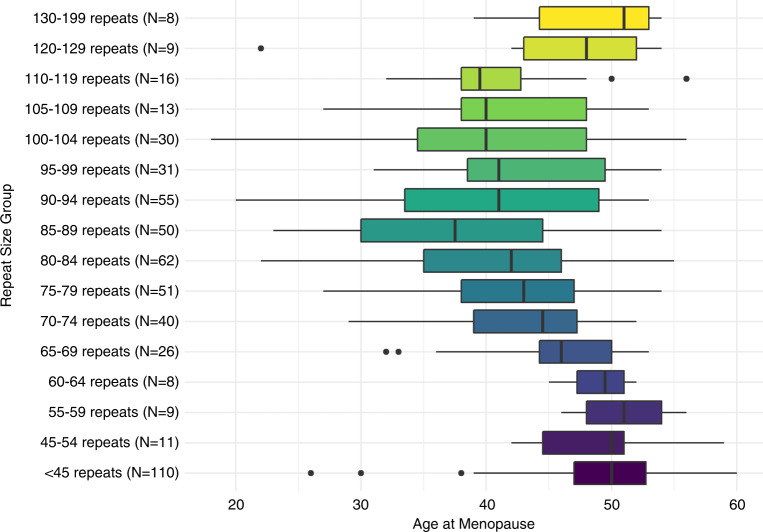
Box plot of age at menopause distribution by repeat size group. Vertical lines within the box from left to right represent the lower quartile, the median, and the upper quartile, respectively. The horizontal lines represent the 5th and 95th percentiles, and the values beyond these lines, marked as dots, are considered outliers.

To test for differences in average AAM across repeat groups, we first used an ANOVA model that was adjusted for age at interview (*p* < 0.0001), race/ethnicity (*p* = 0.88), smoking pack years (*p* = 0.0006), and BMI (0.99). Only the 529 women with an assigned AAM were included in this analysis. Table 2 shows the adjusted least squares means with the 95% confidence limits. Figure 1 shows the distribution of raw AAM for each of the repeat size groups. The sample size for each repeat size group in the model is also shown. Groups shown in bold in Table 2 were significantly different from the referent group (<45 repeats) using Tukey’s Studentized range test that adjusts for multiple testing. Importantly, repeat size groups with <70 repeats and ≥120 repeats were not significantly different from the referent group.

**Table 2:** Outcome measures by repeat size group for the three statistical models used to evaluate age at menopause (AAM) or risk of FXPOI.

	ANOVA (*N* = 529)^a,b^	Logistic Regression (*N* = 903)^a^	Survival Analysis (*N* = 1,607)^b^
Repeat Group	Least squares mean for AAM (95% CI)	OR for FXPOI (95% CI)	HR for Menopause (95% CI)
<45 repeats (ref)	47.8 (46.6–49.1)	-	-
45–54 repeats	48.2 (44.4–52.1)	N/A^c^	1.2 (0.7–2.3)
55–59 repeats	50.2 (46.0–54.5)	N/A^c^	1.0 (0.5–1.9)
60–64 repeats	47.2 (42.7–51.7)	N/A^c^	1.5 (0.7–3.2)
65–69 repeats	44.5 (42.0–47.0)	**6.4 (1.3**–**30.1)**	**3.6 (2.3**–**5.6)**
70–74 repeats	**43.1 (41.1**–**45.1)**	**20.2 (6.1**–**67.0)**	**4.6 (3.2**–**6.6)**
75–79 repeats	**42.7 (41.0**–**44.5)**	**23.1 (7.4**–**72.3)**	**4.0 (2.8**–**5.6)**
80–84 repeats	**40.6 (39.0**–**42.2)**	**41.7 (13.7**–**126.8)**	**4.6 (3.3**–**6.3)**
85–89 repeats	**38.7 (36.9**–**40.5)**	**37.7 (12.4**–**114.5)**	**5.2 (3.7**–**7.3)**
90–94 repeats	**41.4 (39.7**–**43.1)**	**29.9 (9.8**–**91.0)**	**4.4 (3.2**–**6.2)**
95–99 repeats	**43.3 (41.0**–**45.5)**	**14.2 (4.2**–**48.4)**	**3.0 (2.0**–**4.6)**
100–104 repeats	**41.7 (39.3**–**44.0)**	**30.3 (9.2**–**100.0)**	**2.8 (1.9**–**4.3)**
105–109 repeats	**41.2 (37.7**–**44.7)**	**32.2 (7.5**–**139.1)**	**4.6 (2.6**–**8.2)**
110–119 repeats	**41.9 (38.7**–**45.1)**	**24.2 (6.5**–**90.5)**	**3.9 (2.3**–**6.5)**
120–129 repeats	44.6 (40.4–48.8)	2.9 (0.3–28.2)	2.0 (1.0–4.0)
130–199 repeats	48.4 (43.9–52.8)	3.7 (0.4–36.4)	1.6 (0.8–3.4)

All models were adjusted for age at interview, smoking pack years, race, and BMI. Bold indicates a significant difference (<0.05) from the <45 repeats (referent) group. Deepness of color indicates variation of the test statistic across repeat groups.

^a^Age at interview (*p* < 0.0001).

^b^Smoking pack years (*p* < 0.001) significant in model.

^c^Odds ratio could not be calculated because there were no women with POI in that repeat size range.

#### Adjusted odds ratios (OR) for FXPOI per repeat size group (logistic regression)

**Fig. 2:**
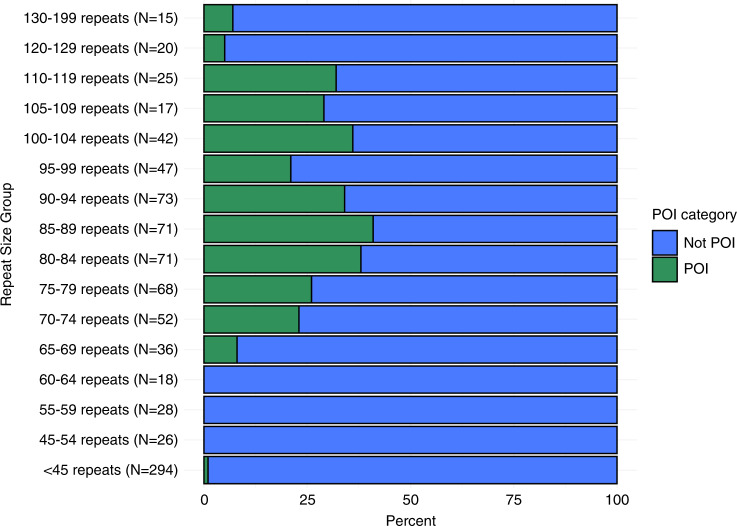
Risk of POI by repeat size group. Raw percentages of women with fragile X–associated primary ovarian insufficiency (FXPOI) (age at menopause [AAM] < age 40) by repeat size group.

Using a logistic regression model with a binary variable for POI included a total of 903 women. This model was also adjusted for age at interview (*p* < 0.0001), race/ethnicity (*p* = 0.10), smoking pack years (*p* = 0.18), and BMI (*p* = 0.68). Figure 2 shows the raw percentages of POI for each repeat size group, and Table 2 shows the odds ratios and 95% confidence interval for each repeat size group from the adjusted model. The odds ratios that are significantly different from referent group are shown in bold in Table 2. For this analysis, repeat groups with <65 repeats and ≥120 repeats were not significantly different from the referent group. Odds ratios could not be calculated for repeat groups with 45–64 repeats because there were no women with POI in that repeat size range.

#### Adjusted hazard ratios (HR) for FXPOI per repeat size group (survival analysis)

**Fig. 3:**
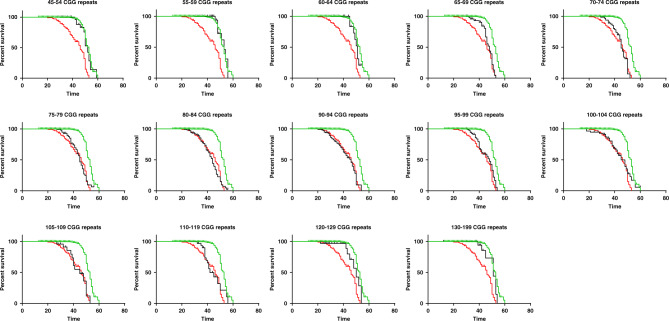
Survival analysis plots for each repeat size group. Survival analysis plots are shown for each repeat size group (shown in black) compared to the low-risk <45 repeats group (shown in green) and the high-risk group of 85–90 repeats (shown in red).

Using survival analysis enables us to include the information from the maximum number of women: a total of 1,607 women were included in this model. As described above for the ANOVA model, 529 women had an assigned AAM, and the remaining 1,078 women were censored from the analysis at the age described in statistical methods. The model was adjusted for age at interview (*p* = 0.001), race/ethnicity (*p* = 0.61), smoking pack years (*p* < 0.0001), and BMI (0.24). Consistent with the ANOVA and logistic regression models, repeat groups with <65 repeats and ≥120 repeats were not significantly different from the referent group (Table 2). Figure 3 shows survival curves for each repeat size group (shown in black) compared to the low-risk <45 repeats group (shown in green) and the highest risk group (85–89 repeats; shown in red). The 85–89 repeat size group was chosen as the high-risk group because this group showed the lowest average AAM. The difference in survival curves reflect the findings for the hazard ratios presented in Table 2.

### Cycling traits based on repeat size groups

We also examined self-reported cycling traits for each repeat size group. Women were asked to describe these traits for the last year they were naturally cycling. No significant difference in reported age at menarche was detected between groups in an ANOVA model that was adjusted for age at interview and race/ethnicity (Supplementary Table 1). The remaining cycling traits were each examined using logistic regression models that were adjusted for age at interview, race/ethnicity, smoking pack years, and BMI. Reporting of short cycle length, defined as <27 days, was significantly higher in the 70–74 repeat size group (OR = 1.77; 95% CI = 1.04–3.02) and the 90–94 repeat size group (OR = 1.57; 95% CI = 1.02–2.42). Short bleed length was defined as <5 days of bleeding, and the 80–84, 85–89, 95–99, and the 105–109 repeat group showed an increased likelihood of reporting this (OR = 2.22, 95% CI = 1.42–3.45; OR = 1.83, 95% CI = 1.17–2.82; OR = 1.68, 95% CI = 1.01–2.74; and OR = 3.19, 95% CI = 1.38–7.35, respectively). Two measures of cycle irregularity were also included. For the first variable of irregular cycles, four repeat groups reported significantly increased frequencies compared to the referent group: 70–74 repeats (OR = 1.70, 95% CI = 1.01–2.85), 85–89 repeats (OR = 1.94, 95% CI = 1.28–2.93), 90–94 repeats (OR = 1.88, 95% CI = 1.24–2.83), and 120–129 repeats (OR = 2.26, 95% CI = 1.09–4.77). The second variable for irregularity asked if women ever went six weeks or more without having a period, and the majority of repeat groups between 75 and 104 repeats reported a significantly increased likelihood of this compared to the referent group (Supplementary Table 1).

### Fertility traits based on repeat size groups

Two variables were analyzed to investigate the presence of fertility issues: (1) going a year or more having unprotected intercourse without getting pregnant and (2) visiting a doctor, clinic, or hospital because they could not get pregnant. Logistic regression was used to test for the likelihood of reporting these variables compared to the referent group. Four repeat size groups showed an increased likelihood of ever going a year or more having unprotected intercourse without getting pregnant compared to the <45 repeats group: 75–79, 85–89, 90–94, and 110–119 repeats (Supplementary Table 2). The second question, if they had ever visited a doctor, clinic, or hospital because they could not get pregnant, indicated a significantly increased OR for repeat groups from 75–94 repeats (Supplementary Table 2).

### Repeat size mosaicism as a possible modifier of risk

Mailick et al.^34^ found that women with a premutation allele with PM/FM mosaicism of their repeat size, as measured in blood, were associated with better health, mental health, and executive function compared with women who were nonmosaic for a premutation allele. Based on these findings, we evaluated whether repeat size mosaicism altered the risk of FXPOI in our population. We had information on the presence of mosaicism on 320 women with a premutation (Asuragen assay conducted; see “Materials and Methods”). Based on this assay, 91 women were identified to have PM/PM mosaicism and 7 women had PM/FM mosaicism. We performed a survival analysis to identify whether either type of mosaicism had a significant impact on AAM. This model included the presence of mosaicism as the predictor variable, adjusted for repeat size and the quadratic of repeat size. The presence of PM/PM mosaicism and of PM/FM mosaicism were each tested separately using a binary variable (1 = mosaicism was present, 0 = no mosaicism detected). Neither type of mosaicism was significant in the models tested; although, the limited sample size for PM/FM mosaicism was clearly a limitation. The distributions for repeat size by AAM on the 131 women with an assigned AAM by presence of type of mosaicism as assessed by the Asuragen assay are shown in Supplementary Figure 1.

## DISCUSSION

Using a cross-sectional study of reproductive histories from 1,668 women, we have detailed our findings on risk for FXPOI and associated traits, including decreased AAM, stratified by high-resolution repeat size groups to provide more personalized risk information for women with a premutation. Using multiple statistical methods, we found consistent results with previous studies:^10,22–26^ women with midrange premutation repeats were at the highest risk for FXPOI and earlier AAM. Because of this large sample, we were able to garner additional risk information using more detailed assessments. In general, women with a repeat size of 70–120 experience a 5-year reduction in AAM—a difference of 5 years in this window of fertility has important implications for family planning and overall health. Earlier onset for ovarian insufficiency has a strong negative impact on a woman’s overall reproductive, physical, and mental health. Beyond the significant consequences of infertility and not being able to have children, women with POI also have earlier onset of bone loss, cardiovascular disease, and menopausal symptoms of hot flushes and night sweats, which can severely impact quality of life. Significantly, women with 85–89 repeats have the highest risk for ovarian insufficiency: they had an average AAM ~10 years earlier than the <45 repeats group. This compares to women in the surrounding repeat intervals of 80–84 repeats and 90–94 repeats who had a 7-year reduction, and, moving out an additional repeat interval, women with 75–79 or with 95–99 repeats who had an average AAM that was 5 years earlier than the referent group (Supplementary Table 3). The percentage of women reporting POI (AAM < 40) or early menopause (AAM < 45) shows a similar pattern (Supplementary Table 3). Consistent with our previous work,^10^ we were also able to detect altered cycling characteristics and fertility traits among women with the midrange of repeats during their last year of natural cycling. Understanding why this narrow peak of repeat sizes imposes such a high risk should provide insight into the etiological mechanism.

Importantly, women with 55–64 repeats did not show an increased risk in any of the models that were run, and in fact, there were not any women in this repeat range that reported their AAM as less than age 45 (Supplementary Table 3). To ensure the lack of significance was not related to the reduced sample size in these groups, we combined the two categories for a total of 88 women, and the findings were consistent: women with 55 to 64 CGG repeats do not show an increased risk for an earlier AAM or FXPOI compared to the referent group. Further, women within this repeat size range did not report an increase in altered cycle traits or fertility issues. This finding has important implications for women who are identified as having a premutation allele through population screening, as the majority of these women fall within this smaller premutation repeat size range.^14,19–21,35^

Of note, the repeat group with 45–54 repeats, those with intermediate alleles, did not have any significantly different findings compared to the <45 repeat referent group (Table 2, Figs. 1–3). Previous studies on this repeat range have reported conflicting results;^36–38^ however, in our population we do not see evidence for an earlier age at menopause in the 45–54 repeat group.

Based on the work by Mailick et al.,^34^ we hypothesized that presence of a PM/FM mosaic pattern in women may explain why women with higher premutation repeats have a decreased risk for FXPOI. Unfortunately, our sample size of women with PM/FM was not sufficient to test this hypothesis, as only two of the seven PM/FM mosaic women had an assigned AAM. Both women with PM/FM mosaicism had AAM over age 50; however, this was similar to the others in the same high premutation range (>120 repeats) (Supplemental Fig. 1). A larger cohort or a collaborative effort to target women with a PM/FM mosaic profile would be needed to test this hypothesis.

The primary limitation of this work is that the data are largely based on self-report through a structured questionnaire; although, for a subset of the data, a clinician (H.S.H.) conducted qualitative interviews. Further, women completed interviews at different time points in their reproductive lifespan: some women were still cycling while others had gone through menopause years before they completed the interview. In addition, women who have experienced POI or infertility may have a greater motivation to participate in research. Importantly, these limitations of the data apply to all repeat size groups.

In conclusion, we have used our cohort of women to determine more accurate risk guidelines for FXPOI based on repeat size groups. An important next step for this work is to ensure this information is available to women with a premutation. Providing more specific risk rates for FXPOI for women who are identified through screening studies has the potential to lessen the psychological burden that comes with this diagnosis. Qualitative data from women with a premutation have identified a desire for women to understand their risk for FXPOI.^14,15,17,39^ A lack of educational materials and clinician training might be contributing to the difficulty women experience when they receive a FXPOI diagnosis.^13,39,40^ In many cases, a genetic counselor is the essential health-care professional to work with these families from precounseling to diagnosis to postcounseling.^16^ Thus, for our next steps, we will work with genetic counselors to study the best methods for distributing this more accurate FXPOI risk information to families as well as health-care professionals.

## Supplementary information

Supplementary All

## Data Availability

All data described here will be made available upon request from the corresponding author.
