# Supplementary material for: Refining the risk for fragile X–associated primary ovarian insufficiency (FXPOI) by FMR1 CGG repeat size
Source: Genet Med. 2021 Apr 29;23(9):1648–55. doi: 10.1038/s41436-021-01177-y (PMC8460441; doi:10.1038/s41436-021-01177-y)
Supplement: Supplementary file 1 — Supplementary All [file 41436_2021_1177_MOESM1_ESM.pdf]

Supplementary Table 1. Cycling characteristics. Mean age at menarche and crude percentages for reporting of cycling traits are presented. Odds ratios from logistic regression models comparing the reporting of cycling traits are shown with significant differences from the <45 repeat group shown in bold.

Repeat Group	N	Menarche[A] Mean ± S.E.	Short cycle[B] (<27 days) %		Short bleed length[B] (<5 days) %		Irregular cycles[B] (±2 days) %		Skipped cycles[B] (>6 weeks) %	
			Crude %	OR (95% CI)	Crude %	OR (95% CI)	Crude %	OR (95% CI)	Crude %	OR (95% CI)
<45 repeats	634	12.4 ± 0.1	27.8	-	24.5	-	33.3	-	23.2	-
45-54 repeats	66	12.7 ± 0.2	25.8	0.90 (0.49-1.63)	23.8	0.95 (0.50-1.70)	46.0	1.80 (1.05-3.04)	25.4	1.20 (0.64-2.16)
55-59 repeats	50	12.3 ± 0.2	37.0	1.58 (0.84-2.96)	20.8	0.82 (0.38-1.63)	40.4	1.32 (0.70-2.41)	21.3	0.87 (0.40-1.75)
60-64 repeats	38	12.6 ± 0.2	40.5	1.80 (0.91-3.56)	32.4	1.54 (0.73-3.08)	43.2	1.41 (0.71-2.76)	32.4	1.43 (0.67-2.87)
65-69 repeats	53	12.2 ± 0.2	22.4	0.76 (0.38-1.53)	34.7	1.74 (0.92-3.21)	40.8	1.22 (0.66-2.21)	37.5	1.68 (0.89-3.09)
70-74 repeats	73	12.4 ± 0.2	40.0	**1.77 (1.04-3.02)**	31.3	1.47 (0.83-2.53)	48.5	**1.70 (1.01-2.85)**	23.9	0.90 (0.48-1.60)
75-79 repeats	112	12.4 ± 0.1	32.6	1.29 (0.81-2.05)	31.3	1.45 (0.90-2.30)	41.8	1.34 (0.86-2.08)	35.0	**1.64 (1.03-2.57)**
80-84 repeats	110	12.4 ± 0.1	28.0	1.01 (0.63-1.63)	41.0	**2.22 (1.42-3.45)**	41.6	1.33 (0.86-2.06)	42.2	**2.20 (1.41-3.42)**
85-89 repeats	117	12.4 ± 0.1	34.6	1.45 (0.93-2.26)	37.1	**1.83 (1.17-2.82)**	49.5	**1.94 (1.28-2.93)**	38.0	**1.94 (1.25-2.98)**
90-94 repeats	119	12.5 ± 0.1	37.0	**1.57 (1.02-2.42)**	23.6	0.98 (0.60-1.57)	50.0	**1.88 (1.24-2.83)**	35.7	**1.70 (1.09-2.61)**
95-99 repeats	89	12.1 ± 0.2	35.8	1.50 (0.92-2.45)	34.6	**1.68 (1.01-2.74)**	45.1	1.54 (0.96-2.47)	30.9	1.38 (0.82-2.29)
100-104 repeats	62	12.4 ± 0.2	32.2	1.30 (0.73-2.31)	28.3	1.25 (0.68-2.24)	45.0	1.52 (0.88-2.60)	36.7	**1.78 (1.00-3.10)**
105-109 repeats	28	12.9 ± 0.4	29.2	1.12 (0.45-2.77)	50.0	**3.19 (1.38-7.35)**	50.0	1.81 (0.81-4.03)	34.6	1.54 (0.64-3.50)
110-119 repeats	45	12.4 ± 0.2	25.0	0.88 (0.43-1.79)	25.0	1.05 (0.49-2.07)	45.4	1.61 (0.86-3.00)	29.5	1.34 (0.66-2.59)
120-129 repeats	35	12.3 ± 0.2	33.3	1.36 (0.62-2.98)	25.0	1.06 (0.44-2.33)	54.8	**2.26 (1.09-4.77)**	38.7	1.95 (0.89-4.09)
130-199 repeats	37	12.3 ± 0.2	27.8	1.06 (0.50-2.25)	36.1	1.77 (0.85-3.55)	41.7	1.37 (0.67-2.71)	25.0	1.09 (0.47-2.30)

[A] For women with multiple questionnaires, answer from first questionnaire was used

[B] For women with multiple questionnaires, answer from questionnaire closest to conclusion of cycling (see Methods for more details)

All models are adjusted for age when cycling traits are being reported, race/ethnicity, smoking pack years, and BMI.

Supplementary Table 2. Fertility characteristics. Crude percentages for reporting of cycling traits are presented. Odds ratios from logistic regression models comparing the reporting of fertility traits are shown with significant differences from the <45 repeats group shown in bold.

Repeat Group	>1 year of unprotected intercourse w/out pregnancy		Visit a doctor for fertility reasons	
	Crude %	OR (95% CI)	Crude %	OR (95% CI)
<45 repeats	29.8	-	12.2	-
45-54 repeats	30.2	1.03 (0.57-1.81)	14.3	1.23 (0.55-2.50)
55-59 repeats	31.2	1.16 (0.60-2.16)	16.7	1.49 (0.65-3.07)
60-64 repeats	37.8	1.44 (0.69-2.88)	18.9	1.61 (0.63-3.62)
65-69 repeats	28.6	0.96 (0.49-1.80)	16.3	1.48 (0.65-3.06)
70-74 repeats	34.3	1.18 (0.68-2.01)	17.9	1.42 (0.69-2.70)
75-79 repeats	47.1	**2.25 (1.47-3.44)**	22.0	**1.88 (1.09-3.14)**
80-84 repeats	34.0	1.22 (0.77-1.90)	25.5	**2.32 (1.37-3.83)**
85-89 repeats	43.5	**1.75 (1.15-2.65)**	25.9	**2.32 (1.40-3.77)**
90-94 repeats	40.7	**1.67 (1.10-2.54)**	23.0	**1.99 (1.18-3.25**)
95-99 repeats	33.3	1.28 (0.77-2.09)	14.8	1.19 (0.59-2.23)
100-104 repeats	28.3	1.06 (0.57-1.89)	16.7	1.34 (0.61-2.65)
105-109 repeats	34.6	1.37 (0.57-3.09)	28.0	2.49 (0.94-5.97)
110-119 repeats	43.2	**1.98 (1.05-3.70)**	22.7	2.13 (0.96-4.37)
120-129 repeats	34.4	1.29 (0.59-2.66)	12.5	0.89 (0.26-2.33)
130-199 repeats	19.4	0.68 (0.27-1.52)	5.6	0.42 (0.07-1.41)

For women with multiple questionnaires, answer from questionnaire closest to time after reporting of symptoms was used. All models are adjusted for age at interview, race/ethnicity, smoking pack years, and BMI.

Supplementary Table 3. ANOVA model for age at menopause (AAM) by repeat size group with comparison of the number of years earlier AAM occurs for each repeat size group compared to women with <45 repeats.

Repeat Group	ANOVA (N=520): Least squares mean for AAM (95% CI)	Number of years earlier for AAM compared to women with <45 repeats	% of women with POI (AAM<40)	% of women with early menopause (AAM<45)
<45 repeats (ref)	47.8 (46.6-49.1)	-	1.3%	4.9%
45-54 repeats	48.2 (44.4-52.1)	-0.4	0%	12.5%
55-59 repeats	50.2 (46.0-54.5)	-2.4	0%	0%
60-64 repeats	47.2 (42.7-51.7)	0.6	0%	0%
65-69 repeats	44.5 (42.0-47.0)	3.3	7.7%	23.3%
70-74 repeats	**43.1 (41.1-45.1)**	4.7	22.2%	44.4%
75-79 repeats	**42.7 (41.0-44.5)**	5.1	27.0%	46.4%
80-84 repeats	**40.6 (39.0-42.2)**	7.2	39.5%	62.9%
85-89 repeats	**38.7 (36.9-40.5)**	9.1	42.5%	61.9%
90-94 repeats	**41.4 (39.7-43.1)**	6.4	35.1%	50.0%
95-99 repeats	**43.3 (41.0-45.5)**	4.5	23.5%	53.8%
100-104 repeats	**41.7 (39.3-44.0)**	6.1	34.8%	52.8%
105-109 repeats	**41.2 (37.7-44.7)**	6.6	29.4%	50.0%
110-119 repeats	**41.9 (38.7-45.1)**	5.9	30.8%	63.2%
120-129 repeats	44.6 (40.4-48.8)	3.2	4.8%	25.0%
130-199 repeats	48.4 (43.9-52.8)	-0.6	6.2%	22.2%

Bold indicates a significant differences compared to the <45 repeats group.

Supplementary Figure 1. Distribution of age at menopause (aam) by repeat size for women who were assayed for mosaicism. Women with no mosaicism detected are shown in blue, women with premutation/premutation (PM/PM) allele mosaicism (i.e., more than one premutation sized allele detected) are shown in green, and women with premutation/full mutation mosaicism (i.e., women with premutation and full mutation sized alleles detected) are shown in red.

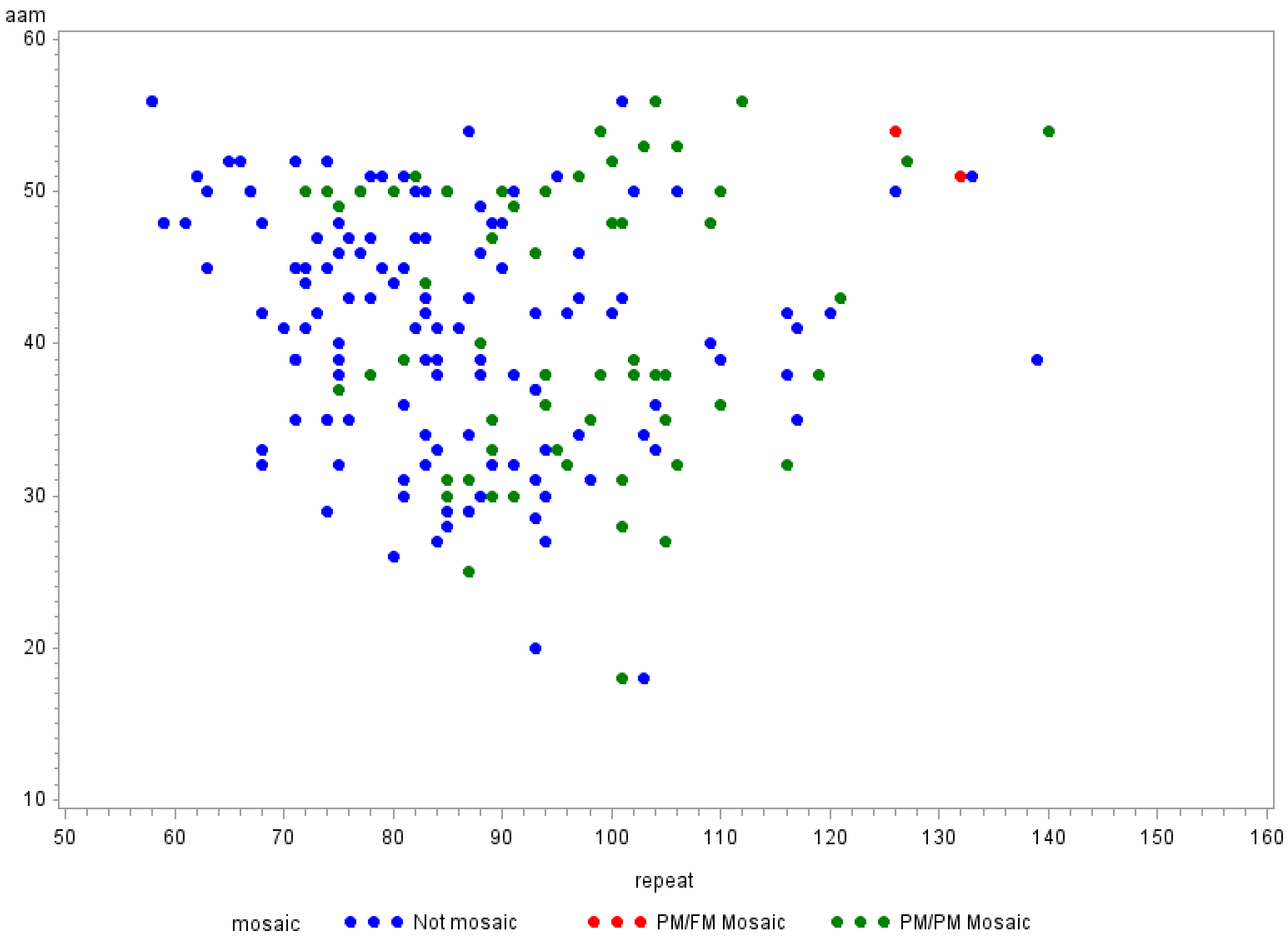
aam
60
50
40
30
20
10
50
60
70
80
90
100
110
120
130
140
150
160
repeat
mosaic
Not mosaic
PM/FM Mosaic
PM/PM Mosaic
